# The Relationship of Spiritual Well-Being and Involvement with Depression and Perceived Stress in Korean Nursing Students

**DOI:** 10.5539/gjhs.v6n4p169

**Published:** 2014-04-14

**Authors:** Younkyung Lee

**Affiliations:** 1 School of School of Nursing, Kimcheon College, Kimcheon-Si, Gyeongsangbuk-Do, Republic of Korea

**Keywords:** depression, spirituality, students, stress (psychological)

## Abstract

This study was conducted to identify the relationship among spiritual well-being, depression and perceived stress. Participants were 518 nursing students located in K province, Korea. Design: Exploratory design was used for this study. Data were collected and analyzed by t-test, ANOVA, Pearson correlation coefficients. The results were as follows; 1) Participants’ mean scores were Spiritual Well-Being 76.03 (15.74), Religious Well-Being 32.8 (15.74), Existential Well-Being 43.23 (8.12), depression 9.10 (7.06), and level of stress 15.47 (5.49). 2) Spiritual Well-Being, Existential Well-Being, and Religious Well-Being were significantly different with the number of attendance to religious ceremony, the degree of subjective satisfaction regard to major and idea for future employment. 3) Negative correlations existed between Spiritual Well-Being and participants’ perceived stress, and depression. 4) Particularly, Existential Well-Being has more inverse correlation with depression and stress than Religious Well-Being. This investigation highlighted Existential Well-Being as an important factor with lower levels of depression and perceived stress. According to these result, Spiritual Well-Being promotion program is needed to develop as a positive concept to adjust college life and spiritual well-being is required to take care of patients as potential power for nursing students in their future.

## 1. Introduction

Korean nursing students can face heavy stress from academic load, clinical practice, license examination, and concern for employment. Excessive stress cause student difficulty in academic performance and adaption for the clinical circumstance needed for interpersonal relationship (Hill & Pargament, 2003), and the most frequently reported obstacle to academic performance in stress, which outranks viral infections, sleep disturbances, concerns about family members and friends, and relationship problems (American College Association, 2003, 2009).

Moreover, previous research found the evidence indicating that an association exists between the experience of stress and subsequent risk major depressive episodes in adults (Hammen, 2005; Kendler, Karkowski, & Prescott, 1999; Kessler, 1997). Depression can be related to negative psychosocial dimension according to previous researches. For example, signs and symptoms of depression include weight loss, fatigue, insomnia, feeling of helplessness, hopelessness and guilt (Wilson et al., 2006). According to a research, depression of Korean college students is serious, and depression in college is worse than that of middle/high school students (Lee, 2004). In 2012, 49.5% of Korean adolescence experienced heavy stress, 30.5% felt depressed more than 2 weeks within the last year, 18.3% seriously considered attempting suicide, and 4.1% actually attempted suicide at least once during the previous school year (Korean Youth Risk Behavior Survey, 2012). Thus, the students and their social support systems should be aware of stress and depression management to address social issues and ensure a successful college life.

Meanwhile, other research has supported that a protective association exists between religiosity or spirituality and negative health outcomes among young people (Cotton et al., 2006). A recent study has suggested that spirituality can be beneficial for the quality of life and well-being for physical and mental health (Sesana, 2006). However, limited research has explored the relationship between mental health and religiosity or spirituality and most research related to health measures religiosity more than spirituality (Williams & Sternthal, 2007).

In fact, Spiritual Well-Being (SWB) or spirituality, abstract concept was conceptualized by Paloutzian and Ellision (1982), is not defined by religious and cultural boundaries. Concretely, spirituality is not the same as religion; one’s spirituality may be expressed through religion, and religion may one’s source of SWB (Fehring et al., 1997; Mauk & Schmidt, 2004). The idea that health includes physical, emotional, psychosocial, environmental, social, and spiritual components is not strange any more. However, systemic reviews of the empirical literature point that religion and spirituality represent understudied variables in health-related research (Rew & Wong, 2006). Health promotion has not made an effort to address spiritual health as an important dimension of holistic health as well. In addition, current research supports that nurses have a high regard for spiritual care. However, clinical nurses tend to serve insufficient spiritual care for lack of systemic education about spirituality at nursing school (Baldacchino, 2008; Koh, 2003). Research regarding nurses’ spiritual care in nursing has shown that nurses with higher personal sense of spiritual well-being have more positive attitudes toward spiritual care (Musgrave & McFarlane, 2004; Stranahan, 2001). Consequently, enhancing personal sense of spiritual well-being for nursing students is considered important educational strategy for holistic approach in the future. Therefore, in the present study, Korean nursing students’ spiritual well-being was measured, and identified the relationship with depression and stress to prepare basic data for efficient spiritual educational program.

The purpose of this study was fourfold; 1) to examine participants’ spiritual well being, depression, stress; 2) to verify personal characteristics related to specific dimension of SWB (Religious Well-Being: RWB or Existential Well-Being: EWB); 3) to identify the relationship between SWB, depression and perceived stress; 4) whether specific dimension of SWB relate to reduced perceived stress and depression.

## 2. Method

### 2.1 Study Design

Exploratory design was used for this study.

### 2.2 Study Sample

Participants included 518 nursing students of college located in K province, Korea. Data was collected from September, 9th to 24th 2013. Participants completed the study voluntarily and they were assured of their anonymity.

### 2.3 Measurement

#### 2.3.1 Spiritual Well-Being

In present study, the Spiritual Well-Being Scale (SWBS) translated into Korean was used (Ha, Han & Choi, 1998) SWBS (Harrington et al., 1990; Musgrave & McFarlane, 2004) includes 20 items, and participants respond on a 6-point scale ranging from 1 (strongly disagree) to 6 (strongly agree). This 20-item Likert scale has two subscales: Religious well-being (RWB), and existential well-being (EWB). Ten items (odd number) measure RWB, and 10 items (even number) measure EWB. Eight items (1, 2, 5, 6, 9, 12, 13, 16) are adverse questionnaires. The total score ranges from 20 to 120, and the 2 subscale scores range from 10 to 60. Higher score indicate greater spiritual, religious, or existential well-being, respectively. Cronbach’s α for SWBS in the original Korean version was 0.89, and in the present study, it is 0.92 (RWB= 0.93, EWB= 0.90).

#### 2.3.2 Depression

The Beck Depression Inventory (BDI) by Beck (1961) translated into Korean by Lee and Song (1991) was applied. BDI is a culture-free set of 21 items, and requires self report by participants (Cotton et al, 2006). The total score ranges from 0 to 63. Rating from 0 to 9 are considered in the normal range, 10 to 19 marginal, 20-29 moderate, 30-39 moderate to severe, and above 40 are severe (Cotton et al., 2006). Cronbach’s α for BDI was 0.86 in Beck’s study, and 0.87 in present study.

#### 2.3.3 Stress

The Korean version of Perceived Stress Scale (PSS) by Lee (2005) was used. PSS was developed by Cohen, Kamark and Mermelstein (1983) and the scale was modified simply by factor analysis. Its general and global nature makes it suitable to measure stress without focusing on specific situations (Cohen et al., 1983; Cohen & Williams, 1998). It comprises 10 items that related to frequency of stress in different situations. Responses on the PSS items indicated the frequency with which the participants experienced certain feelings and thoughts during the last month. Participants respond on a 4-point scale ranging from 0 (never) to 4 (very often), and higher scores represent higher level of stress. Positive 4-items (4, 5, 7, 8) were graded adversely. Cronbach’s α for Korean version of PSS was 0.83, and 0.86 in this study.

### 2.4 Data Analysis

Data were analyzed by SPSS 18.0 program. Frequencies, percentages, means, and standard deviations were calculated to summarize the descriptive data. T-test and ANOVA was used to examine the difference level of SWB, depression, and stress. Scheffe test was used for verification among significant results. Pearson’s correlation coefficients were calculated to identify the relationships among the variables.

## 3. Results

### 3.1 Sample Characteristics

The 518 participant data were analyzed after eliminating invalid or incomplete questionnaires. Most participants were female (n=469, 90.5%), and number of male students were 49(9.5%). Participant average age was 21.35. Approximately 60% reported their religion as none; Christian (18%); Catholic (14%); Buddhist (8%); and others (0.4%). Participants reported never (31%); 1 time a month (40%); 2 or 3times a month (16%); 4 times a month (6%); and more than 5 times a month(7%) for the questionnaire of religious activity. More than half the participants (54.2%) satisfy for nursing as a choice for their major, 34.9% response moderate, and 10.8% dissatisfied. Approximately 63% reported optimistic for their future with regard to employment and 4.8% reported pessimistic (Table 1). Participants reported existential well-being (*M*=43.23, *SD*=8.12), religious well-being (*M*=32.8, *SD*=10.85), and total spiritual well-being (*M*=76.03, *SD*=15.74), depression (*M*=9.10, *SD*=7.06), and stress (*M*=15.47, *SD*=5.49) (Table 2).

**Table 1:** Homogeneity of demographic characteristic

Characteristic	Categories	N (%)
**Gender**	Male	469 (90.5)
	Female	49 (9.5)
**Age**		21. 35
**Religion**	None	310 (59.8)
	Christian	95 (18.3)
	Catholic	40 (7.7)
	Buddhist	71 (13.7)
	Others	2 (0.5)
**Attendance**	Never	161 (31.1)
	1/m	205 (39.6)
	2-3/m	83 (16.0)
	4/m	31 (6.0)
	5↑/m	38 (7.3)
**Satisfaction with major**	Satisfaction	281 (54.2)
	Medium	181 (34.9)
	Dissatisfaction	56 (10.9)
**Career opportunity**	Optimistic	325 (62.7)
	Medium	168 (32.5)
	Pessimistic	25 (4.8)

**Table 2:** Level of spiritual well-being, depression, perceived stress

Variables	Subcategories	M (SD)
**SWB**	RWB	32.80 (10.85)
	EWB	43.23 (8.12)
**Depression**		9.10 (7.06)
**Stress**		15.47 (5.49)

### 3.2 Difference Level of SWB, RWB, and EWB according to Demographic Characteristics

There were no significant difference between males and females regarding levels of SWB, EWB, and RWB. However, four demographic variables (religion, attendance, satisfaction with major, subjective idea for career opportunity) were analyzed factors affecting the level of SWB, EWB, and RWB. Religious participants scored significantly higher levels of SWB (t= -9.889, p<0.000), RWB (t= -11.442, p<0.000), and EWB (t= -3.982, p<0.000), than the areligious group. Participants who attend religious ceremony more than four times a month scored higher levels of SWB (F=40.450, p<0.000), EWB (F=69.340, p<0.000), and RWB (F=10.689, p<0.000) than the less attendant groups. Regarding the question with satisfaction with major, the satisfied group scored high levels of SWB (F=45.450, p<0.000), RWB (F=16.669, p<0.000), and EWB (F=44.690, p<0.000) significantly. Optimistic students for their employment in the future also were likely to have high SWB (F=30.525, p<0.000), RWB (F=8.343, p<0.000), and EWB (F=48.298, p<0.000).

**Table 3:** The difference of spiritual well-being according to demographic characteristic

Characteristics	Variable	M (SD)	t/F	p	Scheffe
**Gender**	Male	76.51 (16.68)	.123	0.726	
	Female	75.99 (15.66)			
**Religion**	Religious	83.77 (17.04)	-9.889	0.000	
	Areligious	70.89 (12.47)			
**Attendance**	none	72.70 (13.12)	51.876	0.000	4↑/m
	1-3 / m	82.52 (16.40)			>1-3/m
	4↑/m	96.95 (16.26)			> none
**Satisfaction with major**	Satisfaction	81.00 (15.46)	40.450	0.000	Satisfaction > Medium
	Medium	71.82 (14.26)			> Dissatisfaction
	Dissatisfaction	64.55 (13.80)			
**Career opportunity**	Optimistic	79.92 (15.52)	30.525	0.000	Optimistic >
	Medium	70.01 (13.26)			Medium, Pessimistic
	Pessimistic	65.80 (17.20)			

**Table 4:** The difference of religious well-being according to demographic characteristic

Characteristics	Variable	M (SD)	t/F	p	Scheffe
**Gender**	Male	30.65 (11.79)	.825	0.364	
	Female	33.03 (10.74)			
**Religion**	Religious	38.83 (11.51)	-11.442	0.000	
	Areligious	28.83 (8.32)			
**Attendance**	none	30.32 (8.89)	69.340	0.000	4↑/m
	1-3 / m	38.59 (10.02)			>1-3/m
	4↑/m	48.03 (10.73)			> none
**Satisfaction with major**	Satisfaction	35.10 (11.13)	16.669	0.000	Satisfaction >
	Medium	30.86 (10.21)			Medium,
	Dissatisfaction	27.51 (9.01)			Dissatisfaction
**Career opportunity**	Optimistic	34.28 (11.37)	8.343	0.000	Optimistic >
	Medium	30.35 (9.21)			Medium
	Pessimistic	30.00 (10.94)			

**Table 5:** The difference level of existential well-being according to demographic characteristic

Characteristics	Variable	M(SD)	t/F	p	Scheffe
**Gender**	Male	45.86(9.50)	3.415	0.065	
	Female	42.95(7.93)			
**Religion**	Religious	44.94(8.21)	-3.982	0.034	
	Areligious	42.06(7.88)			
**Attendance**	none	42.38(8.08)	10.689	0.000	4↑/m
	1-3 / m	43.93(8.00)			>1-3/m
	4↑/m	48.92(8.23)			> none
**Satisfaction with major**	Satisfaction	45.90(7.48)	44.690	0.000	Satisfaction >
	Medium	40.96(7.29)			Medium >
	Dissatisfaction	37.07(8.35)			Dissatisfaction
**Career opportunity**	Optimistic	45.63(7.50)	48.298	0.000	Optimistic >
	Medium	39.66(7.30)			Medium,
	Pessimistic	35.80(8.19)			Pessimistic

### 3.3 Correlates of Variables

Table 6 proves association between SWB, RWB, EWB, stress, and depression. SWB was slightly stronger positive relation with RWB(r=.877, p<0.000) than EWB (r=.766, p<0.000) variable in this study. Correlation analyses indicated that higher religious, existential, and spiritual well-being were associated with lower levels of perceived stress and depression.

**Table 6:** Correlation among spiritual well-being, depression, perceived stress

	RWB	EWB	Stress	Depression
**SWB**	.877*	.766*	- .299*	-.394*
**RWB**	-	.363*	- .153*	- .175*
**EWB**		-	- .376*	- .530*
**Stress**			-	-.358*

* *p* < 0.000

## 4. Discussion

The present investigation examined how SWB related to depression and stress, and which dimension is more connected to psychosocial dimension of health.

In relation to the first question, participants’ mean score of SWB, RWB and EWB were 76.03 ± 15.74, 32.8 ± 10.85, and 43.23±8.12 respectively. These scores are recognized moderate level compare to previous studies for Korean nursing students: SWB 68.32±9.38 (Jung & Eun, 2011): 64.85 (Lee, Park, & Son, 2007): 79.52±16.37 (Choi & Kim, 1998). The level of depression (9.10 ± 7.06) and stress (15.47 ± 5.49) perceived by participant were moderate as other Korean nursing students.

For the second and third questionnaires, religious participants have higher level of SWB, RWB, and EWB than areligious group, and attendance was confirmed factors related to three variables. Participants who were satisfied with nursing department and students positive for employment in their future have higher SWB, RWB, and EWB than others. However, there were no significant differences between male and female.

These findings support basic concept of SWB by Ellison and Palouzian (1982) that SWB as two dimensions: EWB and RWB. EWB refers to the horizontal dimension of having a sense of meaning and purpose in life while RWB indicates the vertical dimension of having a focus on one’s relationship with God or a stronger power. Therefore, religion may affect one’s source for SWB, spirituality is not the same as religion though (Ellison & Palouzian, 1982). However, religion and attendance variables were also related to EWB in present study. This result may suggest that having a religion or attending religious activities might help people to find meaning in their life and existential perception. In fact, previous research proved that involvement in religious activity contributed to increase self-perceptions regarding making sense of life for people with psychiatric disabilities (Sadaaki et al., 2012). As showed in Table 5, EWB has significant relationship with students’ satisfaction regard to major and subjective idea for their future. We may assume that students’ satisfaction and subjective expectation were considered as a significant t part for their existential meaning.

Choi’s (1996) research support these results by proving that nurses who have higher job satisfaction have higher RWB and EWB scores. One study found nurses who have higher RWB scores possess higher hope (Choi & Hur, 1996).

The fourth research questionnaire was that whether specific dimensions of SWB relate to reduced perceived stress and depression. Correlation shows that high level of SWB related to low level of stress and depression, and EWB has more inverse correlation with stress (r= -.376, p<0.000) and depression (r= -.530, p<0.000) than RWB (r= -.153, p<0.000, r= -.175, p<0.000). These findings support previous research. EWB was more negatively correlated depression(r= -.67, p<0.001), hopeless(r= -.66, p<0.001), and suicidal ideation (r= -.56, p<0.001) than RWB (r= -.23, p<0.001, r= -.26, p<0.001, r= -.30, p<0.001) (Taliaferro et al, 2009). Other research investigated showed that EWB had direct additive effect on patients’ with fibromyalgia syndrome perception of uncertainty (Anema et al., 2009). These results may interpret that EWB is more powerful psychosocial internal energy by recognizing one’s existence while SWB is the vertical dimension of having a focus on one’s relationship with God or a stronger power.

Overall, these findings suggest that a strategy for reducing distress and suicidal ideation among college students may involve exploring mechanisms that nurture a sense of meaning in life for individuals whom organized religion remains unimportant (Wright et al., 1993).

There were several studies related to SWB to reduce distress and improve quality of life. One research applied to Mantram repetition over 5 weeks (90 minutes/week) to healthcare workers proved it was significantly effective to reduce stress, trait anxiety and improve quality of life. Mantram repetition was especially significant in its effect to raise EWB (Bormann et al., 2006). Jung and Eun (2011) adopted ASSET (a model for Actioning Spirituality and Spiritual Care Education and Training in Nursing) to Korean nursing students. The model was designed by Narayanasamy (1999) to develop British nurses’ spiritual care and nursing students’ spirituality. Previous studies have supported the model was effective in raising awareness in the importance of spiritual care, and helps to perform spiritual care (Baldacchino, 2008; Pesut, 2002; Shin et al., 2001). Consistent with previous research, ASSET modelling which was applied to Korean nursing student for 6 weeks (2 hours/week) was significant in its effect for spiritual care competence, implementation of spiritual care, personal support and patient counselling, attitude toward the patient’s spirituality, and communication. However, research of development programs for spirituality is not enough, and needs to be studied more.

This study has several limitations. First, this data were collected from one nursing school, so it is needed to be interpreted with caution. Second, this data originated from a cross-sectional survey, so the analyses could not interpret causal relationships and longitudinal studies can help identify cause-and-result relationship.

In spite of these limitations, findings from this study suggest SWB (especially EWB) could be potential power for positive psychosocial dimension. Therefore, we expect new educational program which develop SWB could be helpful for managing students’ stress and depression, and great source for spiritual care in their future.

## 5. Conclusion

In summary, despite some limitations, findings from present study support the contention that SWB, especially as EWB, represents a distinct dimension that adds explanatory power to the prediction of mental health (Hill & Pargament, 2003). However, Korean nursing education system does not recognize the importance of spiritual aspect, and nursing circumstance is still strongly focused on a disease or cure-oriented model. Therefore, overcoming barriers to spiritual health education and research is considered important task, and the results of this study have implications for educational policy and practice.
